# Essential thrombocytosis: diagnosis, differential diagnosis, complications and treatment considerations of relevance for a cardiologist

**DOI:** 10.1007/s12471-023-01757-4

**Published:** 2023-02-09

**Authors:** R. S. Kuipers, L. Kok, R. Virmani, A. Tefferi

**Affiliations:** 1 OLVG Heart Centre, Onze Lieve Vrouwe Gasthuis, Amsterdam, The Netherlands; 2 Department of Cardiology, Dijklander Hospital, Purmerend/Hoorn, The Netherlands; 3 Department of Cardiology, Spaarne Hospital, Haarlem, The Netherlands; 4 CVPath Institute, Gaithersburg, MD USA; 5 Divisions of Hematology and Hematopathology, Mayo Clinic, Rochester, MN USA

**Keywords:** Essential thrombocytosis, Thrombocythaemia, Myeloproliferative, Myocardial infarction

## Abstract

Essential thrombocytosis (ET) is a rare haematological malignancy, with an incidence rate of 1.5–2.5/100,000 per year. For many patients with ET the first manifestation of their underlying disease is a thrombotic or haemorrhagic complication. A recent retrospective study revealed an incidence rate of at least 2.1% in people under 40 years presenting with an acute coronary syndrome, although the diagnosis was initially missed in all cases. Thus, cardiologists face a much higher than average incidence rate of ET in their daily practice, but seem insufficiently aware of the disease. The current review summarises symptoms, (differential) diagnosis, complications and treatment considerations of ET of relevance for a cardiologist. Typical symptoms, besides thrombosis and haemorrhage, include erythromelalgia and aquagenic pruritus, while platelets > 450 × 10^9^/l are a diagnostic for ET once other myeloproliferative neoplasms, secondary and spurious thrombocytosis have been excluded. With regard to treatment, timing of revascularisation depends on the presence of ischaemia and concurrent platelet counts. In the presence of ischaemia, revascularisation should not be delayed and adequate platelet counts can be achieved by platelet apheresis. In the absence of ischaemia, revascularisation can be delayed until adequate platelet counts have been achieved by cytoreductive therapies. Cardiologists should be aware of/screen for possible ET.

## Introduction

Essential thrombocytosis/thrombocythaemia (ET) has been considered a rare underlying aetiology for acute coronary syndromes (ACS) [1, 2]. However, we recently showed a prevalence of at least 2.1% in a cohort of patients under 40 years that underwent coronary angiography (CAG) in the setting of their first ACS [3]. More importantly, as shown previously [4], this diagnosis was either missed/severely delayed (average 6 years) despite the presence of elevated thrombocytes (i.e. > 450 × 10^9^/l) upon presentation. This observation might suggest that ET is insufficiently known among cardiologists. The present review aims at summarising the available literature on ET that is relevant, e.g. with regard to diagnosis and treatment, for the cardiologist.

## Definition and epidemiology

ET is a rare chronic myeloid malignancy, having an incidence rate of 1.5–2.5/100,000 per year [5]. Together with polycythaemia vera (PV) and primary myelofibrosis (PMF), ET is one of the three myeloproliferative neoplasms (MPNs) characterised by stem-cell-derived clonal myeloproliferation with mutually exclusive Janus kinase (JAK)2V617F, calreticulin (CALR) or myeloproliferative leukaemia (MPL) mutations [6]. In ET, JAK2V617F mutations occur in about 55%, CALR mutations in 15–24% and MPL mutations in about 4% of cases. The remainder, about 20%, are so-called triple negative [6]. Median age at diagnosis of ET is in the sixth decade of life [5], with less than 20% of patients being diagnosed below age 40 years [7]. From a population point of view, ET is one of the rare (< 1%) pathologies associated with ACS [1]. From a haematological point of view, the incidence of ACS in patients with ET ranges from 2% to 31% in various studies [1, 8–10]. From a cardiologist’s point of view, we recently showed that the prevalence of ET can be at least 2.1% in certain groups [3]. Taking into account that ACS is considered extremely rare in ET patients under 40 years of age [1], the incidence in various older age groups remains to be elucidated.

## Diagnosis, clinical features and differential diagnosis of ET

A diagnosis of ET is based on the criteria in Tab. 1 [6]. Upon diagnosis, most patients report having experienced either no (27%) or aspecific vasomotor symptoms (66%), ranging from abdominal and atypical chest pain, paraesthesia, dysaesthesia and headaches to syncope. More specific symptoms are erythromelalgia, a syndrome consisting of localised painful burning, redness, warmness and congestion in the extremities [10, 11], as well as aquagenic pruritus, which arises after contact with water [12]. Less than half (44%) report previous symptoms related to thrombosis (18%) or haemorrhage (26%). Unfortunately, some patients initially present with severe complications, such as peripheral, pulmonary, portal vein, cerebral or coronary embolisms or haemorrhagic pericardial effusion. Finally, splenomegaly (26%) and hepatomegaly (3%) were noticed in patients with abdominal pain and ET [13, 14]. Increased routine laboratory screening within the general population will increase the incidental finding of thrombocytosis. At present, thrombocytosis is encountered in 1.5–2.2% of people consulting primary care [15].

**Table 1:** Criteria for a diagnosis of essential thrombocytosis (adapted from [6])

*Major criteria*
1. Platelets > 450 × 10^9^/l
2. A characteristic bone marrow biopsy (with megakaryocyte proliferation and loose clusters)
3. Not meeting WHO criteria for other myeloid neoplasms
4. The presence of a JAK2/CALR/MPL mutation
*Minor criterion*
The presence of another clonal marker or no evidence of reactive thrombocytosis
NB: The diagnosis requires all four major or the minor and the first three major criteria

With regard to the differential diagnosis of thrombocytosis, in clinical practice 80–90% of subjects with a platelet count above 450 × 10^9^/l do not have an essential/primary thrombocytosis, but have secondary/reactive thrombocytosis, which is an abnormally high platelet count secondary to underlying events, disease or medication [15, 16] which might be either acute/transient or chronic. In the case of secondary thrombocytosis, the platelet count is rarely > 1000 × 10^9^/l. Second, a peripheral blood smear might differentiate primary from secondary thrombocytosis, since in contrast to secondary thrombocytosis, in which platelets appear normal, giant platelets may be observed in primary thrombocytosis [17]. In contrast to primary thrombocytosis, reactive thrombocytosis rarely results in thrombotic or haemorrhagic events [16]. Another, in fact erroneous, cause of ‘thrombocytosis’ may result from the use of automated analysers. A variety of clinical conditions may result in spuriously raised platelet counts [18, 19] when these small fragments are counted as platelets by the automated analyser. Hence, a peripheral blood smear might also differentiate primary/essential from artefactual/spurious thrombocytosis. Examples are given in Tab. 2.

**Table 2:** Causes of thrombocytosis (adapted.from [18, 19])

Primary or clonal	Secondary or reactive	Artefactual or spurious
Essential thrombocytosis	Acute blood loss	Erythrocyte fragments
Polycythaemia vera	Trauma	Schistocytes
Primary myelofibrosis	Surgery	Microcytosis
Familial/hereditary thrombocytosis	Infectious diseases	Spherocytosis
Chromosome 5q-deletion syndrome	Iron deficiency	Cryoglobulinaemia
Chronic myeloid leukaemia	Asplenia	Neoplastic fragments
Chronic myelomonocytic leukaemia	Malignancy	Bacteria
Atypical chronic myeloid leukaemia	Chronic inflammation^a^	Fungi
Myelodysplastic syndrome	Haemolysis	Lipid droplets^c^
Unclassifiable myeloproliferative neoplasms	Allergic reactions	–
RARS‑T	Exercise	–
POEMS syndrome	Medication^b^	–

*RARS‑T* refractory anaemia with ringed sideroblasts associated with marked thrombocytosis.

*POEMS* acronym for a rare blood disorder with the following signs/symptoms: polyneuropathy, organomegaly, endocrinopathy/oedema, monoclonal protein, skin changes

^a^For example, rheumatic diseases

^b^For example, low-molecular-weight heparin, ceftazidime, clozapine, gemcitabine, non-steroidal anti-inflammatory drugs and steroids

^c^Notably when samples are taken shortly after a meal

## Thrombotic and haemorrhagic complications associated with ET

Overall rates of complications—in various regions/settings and using different definitions of events—during long-term follow-up in patients with ET are relatively high. The incidence of thrombotic complications ranges from 9% to 84% at diagnosis and from 7% to 32% during long-term follow-up. For haemorrhage these rates range from 4% to 63% at diagnosis and from 8% to 14% during follow-up [8, 20, 21]. With regard to arterial events, cerebrovascular events (relative proportion 55–56%) were shown to be more prevalent than either coronary (22–31%) or peripheral (13–22%) embolisms [1, 8, 9]. With regard to recurrence, as many as 34% of patients with prior thrombosis experience a recurrent thrombotic event. The highest risk for recurrent events is observed within the first 2 years after the first thrombotic event and slowly declines thereafter. Antithrombotic therapy reduces the incidence of recurrent events by about 50% [9].

These numbers clearly indicate that thrombotic complications surpass haemorrhagic complications, that both types of events often coincide with the initial diagnosis of ET and that treatment of ET (after diagnosis) markedly reduces, but does not annihilate, the chance of a second event occurring. Moreover, although ET is a haematological diagnosis, these data also imply that other specialists, such as neurologists, cardiologists and vascular surgeons, should play a proactive role in the identification of ET patients and should thus actively screen for, for example, laboratory anomalies or discrepancies between the event and the risk profile of a patient.

Numerically, myocardial infarction is a rare complication of ET with an incidence rate ranging from 2% to 31% within various studies/regions/settings/periods/definitions [1, 8, 10]. Interestingly, ACS in the setting of ET has been observed in the presence and absence of underlying atherosclerosis [22, 23]. Consequently, as supported by several studies [22], even young patients with ET without cardiovascular (CV) risk factors can experience acute, even life-threatening, thrombotic events. A study that specifically focused on patients that were diagnosed with ET at a young age (i.e. a median age of 31 years) showed similar results compared to those in older adults, with arterial events (18%) being more common than venous events (6%), and cerebrovascular events (13%) more likely than coronary or peripheral embolisms (2% each) [7].

## Possible mechanism for complications in ET

**Fig. 1:**
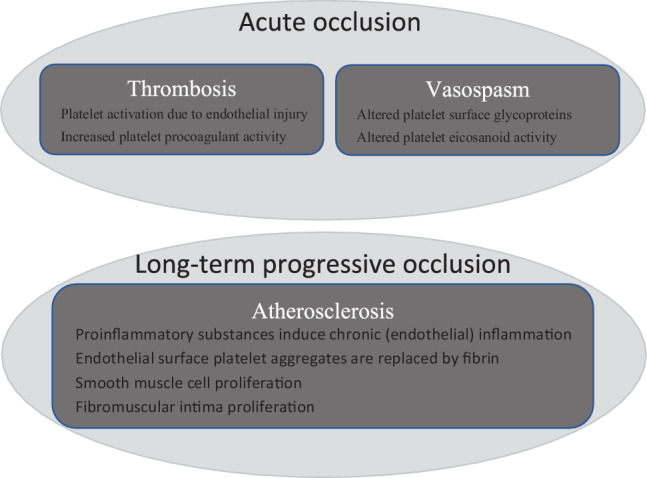
Mechanisms for vaso-occlusive complications in essential thrombocytosis

Both thrombotic and haemorrhagic complications have been observed in the setting of ET. Interestingly, many ET patients that present with an ACS have a normal CAG without signs of atherosclerosis [10, 22–26], which supports the hypothesis that vascular events can be a direct result of the haematological problem, i.e. be unrelated to pre-existing atherosclerosis [27]. In support of this, an > 80% incidence of spontaneous platelet aggregation was shown in patients with ET [28] and large thrombus burden is often described in cases of ACS in the setting of ET (e.g. [29, 30]). Platelet function tests such as prothrombin time, partial thromboplastin time and bleeding time are usually within reference ranges [30]. In those cases where no obstructive coronary artery disease is observed during CAG, it could be argued that thrombus may have resolved after initiation of antiplatelet/anticoagulation therapy combined with delayed (i.e. after several days or months) CAG (e.g. [25]). On the other hand, spasm could be provoked by provocation testing in at least two cases [25, 26], suggesting that besides hyperviscosity, endothelial dysfunction or the release of certain platelet-derived vasospasm-promoting substances, such as serotonin and thromboxane A2, might also contribute to the aetiology of ACS in the setting of ET. In support of this, Cheng and Hung [25] described a patient that experienced recurrent anginal symptoms after discontinuation of diltiazem, but no recurrence of symptoms after discontinuation of cytoreductive therapy despite platelets counts > 900 × 10^9^/l. Both mechanisms for acute occlusion, i.e. thrombotic and vasospastic, and their possible underlying aetiologies are depicted in Fig. 1 [23].

Likewise, alterations in platelet function and composition have been implied in long-term complications of ET (see Fig. 1). For example, organised fibrin, which will replace aggregates of platelets that have become attached to the endothelial surface, may result in extensive intraluminal narrowing of coronary arteries, causing anginal symptoms [27]. Alternatively, the production of proinflammatory eicosanoids [23] and cytokines in the setting of MPNs is thought to explain the development of premature atherosclerosis (and malignancies, see below) secondary to a state of chronic low-grade (endothelial) inflammation in these patients [31]. Finally, other vascular changes, including smooth muscle cell proliferation and fibromuscular intima proliferation, have been described in arterioles as a result of thrombocytosis [11].

## Risk factors for thrombotic and haemorrhagic complications in ET

Age (> 60 years), previous events, the presence of JAK2V617F, leukocytosis and long-term thrombocytosis have been identified as major risk factors for thromboembolic complications [5, 6, 9, 14]. Additionally, the traditional CV risk factors advanced age, smoking, hypertension, hyperlipidaemia and diabetes have been associated with thrombotic complications in the setting of ET [1, 5, 6]. Importantly, the presence of extreme thrombocytosis (platelets > 1000 × 10^9^/l) was associated with a lower risk of thrombosis, possibly through the presence of acquired von Willebrand syndrome (i.e. the structural and/or functional alterations in von Willebrand factor as a result of a concurrent disorder, such as an MPN or, for example, a CV disorder such as aortic stenosis/Heyde syndrome) and consequent increased risk of bleeding [5]. Even at lower rates, platelet number does not seem to be a good predictor of thrombotic events [17]. Also in young patients (< 40 years), cardiovascular risk factors are concurrent stimuli for arterial thrombosis and the use of tobacco was shown to reduce 10-year event-free survival from 90% to 72% [7].

With regard to the risk of recurrent CV events, patients with ET have been stratified into very low-, low-, intermediate- and high-risk individuals (see Tab. 3). Low-risk individuals have an annual risk of thrombosis that is not significantly different from that of the general population at about 0.6–1.3%/year, while the risk increases to 1.8–3.7%/year in the high-risk population [5].

**Table 3:** Risk stratification and preferred treatment options for essential thrombocytosis** (**adapted from [6])

	History of thrombosis	Age > 60 years	JAK2 mutation	CV-RF−	CV-RF+
Very low-risk disease	No	No	No	None	ASA
Low-risk disease	No	No	Yes	ASA q.d.—b.i. d.	ASA b.i. d.
Intermediate-risk disease	No	Yes	No	ASA (± HU)	HU + ASA
High-risk disease	No	Yes	Yes	HU + ASA b.i. d.	HU + ASA b.i. d.
High-risk disease	Yes, arterial	No (or yes)	No (or yes)	HU + ASA b.i. d.	HU + ASA b.i. d.
High-risk disease	Yes, venous	No (or yes)	No	HU + s-ac	HU + s-ac + ASA
High-risk disease	Yes, venous	No (or yes)	Yes	HU + s-ac + ASA b.i. d.	HU + s-ac + ASA b.i. d.

*NB* Treatment with cytoreductive therapy should be avoided in the presence of extreme thrombocytosis (platelets > 1500 × 109/l) and acquired von Willebrand syndrome

*CV-RF* cardiovascular risk factors − (absent), + (present), *ASA* acetylsalicylic acid, *HU* hydroxyurea, *s‑ac* Systemic anticoagulation, *b.i.d.* twice a day

## Haematological complications and mortality in ET patients

ET, similar to the other MPNs, can undergo several transformations. First, transformation of ET into PV has been described in about 2% of patients [32]. Next, both ET and PV can transform/progress into (post-ET/PV) myelofibrosis [33]. In ET, evolution into myelofibrosis occurs in about 3% of patients after 5 years, in 8% after 10 years, and in 15% after 15 years [34]. For PV these rates are slightly higher [35]. Finally, all three MPNs can transform directly, and for ET/PV indirectly via post-ET/PV myelofibrosis, into acute myeloid leukaemia. Overall transformation rates of the three MPNs into leukaemia are in the order of ET (2.6%), PV (3.9%) and PMF (9.3%) in 20 years [36]. Finally, patients with MPNs have a higher risk of various second malignancies, e.g. of the skin, brain, kidney and endocrine organs (odds ratio ≥ 2.5) [32, 36, 37]. As a result, patients with MPNs have reduced survival rates. Five-year survival rates for ET, PV and PMF are 89%, 88% and 45%, respectively [36]. Taken together, all MPNs have now been associated with a slightly reduced life expectancy for which age > 60, leukocytosis, male gender, the presence of concurrent adverse mutations and a history of thrombosis conferred independent risk factors [6].

## ET and pregnancy

Due to abnormal thrombocyte function, pregnancy in the context of MPNs poses unique fetal and maternal challenges. In ET, about 30% of pregnancies are lost, mostly within the first trimester [38]. From a cardiological point of view, a medical history of repeated fetal losses in, notably young, patients with (recurrent) thrombotic events should raise the suspicion of underlying haematological pathology. Hence, taking a gynaecological medical history is warranted in these subjects, since it might add to the understanding of the pathophysiology of thrombotic events in low-risk individuals.

## Treatment of thrombocytosis and prevention of thrombotic events

Treatment of ET should be individualised, bearing in mind all possible complications of ET ranging from thrombotic and haemorrhagic events, the presence of risk factors, and the risk of progression to myelofibrosis or myeloid leukaemia. For clinicians in Europe, the European LeukemiaNET (www.leukemia-net.org) provides guidance based on the accumulating evidence with regard to optimal treatment.

With regard to the prevention of thrombotic complications in ET, the advocated approach [6], based on risk stratification by a history of (arterial or venous) thrombosis, age > 60 years, the presence of a JAK2 mutation and cardiovascular risk factors, is presented in Tab. 3. As an exception, in the case of a definite diagnosis of coronary vasospasm, discontinuation of cytoreductive therapy may be advised in patients below 60 years and in those over 60 years in the absence of a JAK2 mutation and cardiovascular risk factors. Otherwise (see Tab. 3), antiplatelet agents, e.g. acetylsalicylic acid (ASA), are the first-line choice in the prevention of (recurrent) events, since there is little experience with P2Y12 inhibitors [21].

Next in line are cytoreductive therapies, including hydroxyurea (HU), anagrelide and interferon‑α, all of which should be initiated with monitoring of platelet count, erythrocyte and leukocyte levels. Generally, HU is the drug of first choice. In the landmark randomised clinical trial, HU was shown to reduce the risk of thrombotic complications from 10.7% to 1.6% [39]. Due to concerns regarding an increased risk of development of leukaemia during the use of HU, other drugs such as anagrelide were developed. However, in subsequent trials HU + ASA were shown to be superior to anagrelide + ASA with regard to vascular events and transformation rates [40]. For HU, there are no clear contraindications. Interferon‑α is contraindicated in patients with known cardiovascular/thyroid disease. Anagrelide, a phosphodiesterase inhibitor with positive inotropic/chronotropic effects, should be used with caution in patients with known cardiovascular disease, and its use should be accompanied by accurate monitoring of cardiac function and QT interval before and during treatment [20]. Finally, a selective JAK1/JAK2 inhibitor, ruxolitinib, has been developed and has proven its efficacy in patients with both PCV and myelofibrosis, but its use in ET seems limited [6].

## Prevention of recurrent thrombotic events in ET

In a large retrospective study of patients with primary thrombocytosis who received either antiplatelet agents, anticoagulation, cytoreductive treatment or underwent phlebotomy after a first thromboembolic complication, only cytoreductive therapy resulted in a significant reduction in recurrent ACS [9]. Recently, the use of ASA twice daily has been advocated in specific individuals (Tab. 3). An alternative way to prevent recurrent arterial events might be the use of more aggressive, e.g. double antiplatelet, therapy in the first 3–4 years following an arterial thrombotic event [41]. Importantly, antiplatelet therapy plus anticoagulants should be used with caution, since their co-use was shown to result in an almost three times higher incidence of major bleeding as compared to either antiplatelet drugs or anticoagulants alone [9].

## Treatment of the cardiovascular complications of ET

**Fig. 2:**
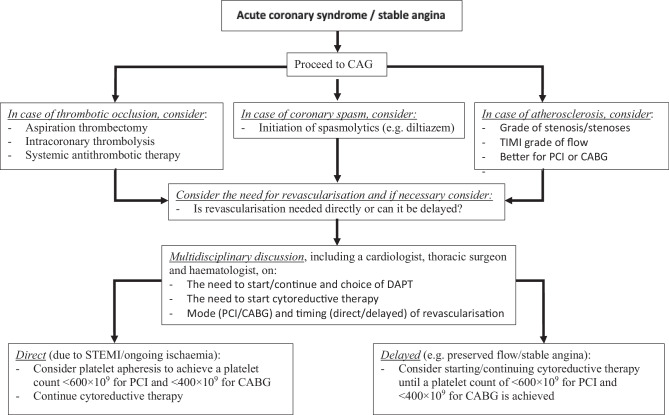
Flow diagram for the treatment of patients with underlying essential thrombocytosis and an acute coronary syndrome or stable angina undergoing coronary angiography (*CAG*). *TIMI* thrombolysis in myocardial infarction,* DAPT* double antiplatelet therapy, *PCI* percutaneous intervention, *CABG* coronary artery bypass grafting,* STEMI* ST-elevation myocardial infarction

With regard to the treatment of ACS in the setting of ET, thrombus aspiration [24, 30], intracoronary thrombolysis [24], balloon angioplasty [30], stent placement [42, 43], coronary bypass grafting (CABG) [44] and systemic fibrinolytic/thrombolytic treatment [10] have all been described. The optimal choice of intervention and the timing thereof should be on an individual basis, taking the presence of spasm, thrombus, atherosclerosis, ongoing ischaemia and concurrent platelet counts into account. In the case of thrombotic occlusions (Fig. 2), besides double antiplatelet therapy, aspiration thrombectomy, intracoronary thrombolysis, antithrombotic therapy with, for example, heparin and/or glycoprotein IIb/IIIa receptor antagonists, and even the use of distal protection devices have all been advocated [24]. Diltiazem was used successfully in a case of coronary vasospasm in the setting of ET [25]. Finally, intracoronary imaging such as intravascular ultrasound or optical coherence tomography could be helpful for evaluating endothelial structure and the presence of atherosclerosis [24].

The main problem with regard to percutaneous coronary interventions (PCIs) in ET patients lies in the choice of antiplatelet regimen due to the high risk of in-stent thrombosis associated with ‘thrombopathy’ [43, 45]. In-stent thromboses have been described in patients with ET after treatment with ASA monotherapy [46], ASA in combination with P2Y12 inhibition [47] and in a patient who underwent primary PCI while having a platelet count of 2100 × 10^9^/l after initiation of ASA, P2Y12 inhibition and cytoreductive therapy [48]. Consequently, although a relation between the absolute platelet count and the risk of thrombosis has been a matter of dispute, delayed stenting (up to several weeks) to initiate (additional) antiplatelet therapy and/or achieve lower platelet counts (i.e. below 400–600 × 10^9^/l) has been advocated [21, 39, 49]. In support of this strategy, several cases with favourable outcomes have been described when stenting of a significant stenosis, with preserved flow (i.e. thrombolysis in myocardial infarction (TIMI) grade 2 or 3), was delayed several weeks to initiate cytoreductive therapy [49], while a case series of 15 did not show an increased risk of complications in PCI patients with an average platelet count of 581 × 10^9^/l [42].

Hence, it has been argued that revascularisation in patients with a platelet count > 400–600 × 10^9^/l should be discussed in a multidisciplinary team, taking both the risk of thrombosis and progressive ischaemia into account (Fig. 2). In the case of ongoing ischaemia, an early invasive intervention should be performed regardless of the patient’s platelet count (see Fig. 2). In such a situation, periprocedural platelet apheresis can provide a rapid and relatively safe reduction of platelets [21]. Apheresis, however, has a short duration of action and acute stent thrombosis has been described in the setting of a PCI that was performed directly after platelet apheresis [48], supporting the notion that cytoreductive therapy needs to be started as soon as possible [21] or apheresis repeated until adequate platelet counts have been achieved.

With regard to surgery, including CABG, reduction of platelet counts to below normal levels (i.e. < 400 × 10^9^/l) with cytoreductive or even platelet apheresis therapy (see Fig. 2) has been advocated in the perioperative setting [21]. Additionally, daily platelet counts are warranted and resumption of cytoreductive therapy is recommended as soon as the patient is able to take oral medication [21]. Conversely, it has been advised that ASA be discontinued a week before surgery if there is a high risk of bleeding or when perioperative anticoagulation is required, but can be restarted 24 h after surgery if no excessive bleeding has occurred or is anticipated [20]. Off-pump procedures have been advocated in cases of haematological disease to avoid adverse effects of cardiopulmonary bypass and surgical complications of extreme bleeding or thrombosis. Finally, bioprostheses should be preferred to avoid lifetime warfarin therapy [50].

## Conclusion

Although ET is a rare haematological malignancy, its prevalence is much higher in patients presenting with either thrombotic or haemorrhaghic events, such as patients seen by a cardiologist, neurologist or vascular surgeon. Any specialist should be aware of such underlying pathology and thus actively screen for elevated thrombocytes (i.e. a platelet count > 450 × 10^9^/l) in patients presenting with either thrombotic or haemorrhaghic events, notably in those without concomitant CV risk factors. If the platelet count continues to be elevated, a haematologist should be consulted and underlying disease should be excluded. Finally, therapeutic options with regard to treatment of ischaemia should be discussed in a multidisciplinary team, including a haematologist, prior to any intervention to prevent complications related to abnormal platelet functions.
